# How researchers can make verbal lie detection more attractive for practitioners

**DOI:** 10.1080/13218719.2022.2035842

**Published:** 2022-03-22

**Authors:** Aldert Vrij, Ronald P. Fisher, Sharon Leal

**Affiliations:** a Department of Psychology, University of Portsmouth, Portsmouth, UK; b Department of Psychology, Florida International University, Miami, FL, USA

**Keywords:** cross-cultural deception, cues of truthfulness, cues to deceit, cues to deceit cross-cultural deception, cues to truthfulness, cut-off points, verbal baselining, verbal lie detection, verbal lie detection cut-off points

## Abstract

Over the last 30 years deception researchers have changed their attention from observing nonverbal behaviour to analysing speech content. However, many practitioners we speak to are reluctant to make the change from nonverbal to verbal lie detection. In this article we present what practitioners believe is problematic about verbal lie detection: the interview style typically used is not suited for verbal lie detection; the most diagnostic verbal cue to deceit (total details) is not suited for lie detection purposes; practitioners are looking for signs of deception but verbal deception researchers are mainly examining cues that indicate truthfulness; cut-off points (decision rules to decide when someone is lying) do not exist; different verbal indicators are required for different types of lie; and verbal veracity indicators may be culturally defined. We discuss how researchers could address these problems.

Over the last thirty years deception researchers have changed their attention from observing nonverbal behaviour to analysing speech content (Vrij, 2019). They have good reason to do so. Meta-analyses of deception research have shown that verbal cues are typically more diagnostic than nonverbal cues to deceit (DePaulo et al., 2003); and that training in verbal lie detection results in a larger improvement in the ability to detect truths and lies than training in nonverbal lie detection (Hauch et al., 2016).

Many practitioners we meet rely on nonverbal cues when they attempt to detect deceit. This could be the result of their training. Police manuals overwhelmingly pay attention to nonverbal cues to deceit (Vrij & Granhag, 2007) and most lie detection training available focuses on nonverbal cues to deceit. Many practitioners we talk to are reluctant to make the change from nonverbal to verbal lie detection. In this article we present what practitioners believe is problematic about verbal lie detection: the interview style typically used is not suited for verbal lie detection; the most diagnostic verbal cue to deceit (total details) is not suited for lie detection purposes; practitioners are looking for signs of deception but verbal deception researchers are mainly examining cues that indicate truthfulness; cut-off points (decision rules to decide when someone is lying) do not exist; different verbal indicators are required for different types of lie; and verbal veracity indicators may be culturally defined. Several of those issues have already been raised by researchers (e.g. Nahari et al., 2019; Vrij, 2016), but the list in this article is more extensive than discussed in previous publications.

We want researchers to address the practitioners’ concerns. Researchers conduct research, in part, because of their curiosity about the underlying theoretical concerns, but also to solve real-world problems (applied research). To solve real-world problems, it is important to come up with ways to address them. Comments sometimes heard amongst verbal deception researchers, such as ‘Verbal lie detection is more accurate than nonverbal lie detection’ and ‘Many of the problems do not just apply to verbal lie detection; they also apply to nonverbal lie detection,’ will not convince practitioners to use verbal lie detection. Instead, more creative arguments are required. In this article we offer such arguments by making suggestions about future research that will address the practitioners’ concerns. Currently, we do not always have appropriate answers for practitioners, but, if we conducted the recommended research, we might have more appropriate responses.

Before we start this article, one limitation merits mentioning. We talked to a limited number of practitioners, and their views may not be representative of all practitioners. To grasp a view that is less anecdotal a very large survey is required. Such a survey could result in more concerns than we report in this article. For example, perhaps more mundane factors explain the reluctance amongst practitioners to make the switch to verbal lie detection, such as a resistance to change what they once learned; disbelief about the validity of novel findings in lie detection; and difficulties in making the required changes in the organisations they are working in. We do not discuss such factors because researchers can only have a limited role to address them.

### The interview style typically used is not suited for verbal lie detection

Probably the four most researched veracity lie detection methods are the Strategic Use of Evidence (SUE), the Verifiability Approach (VA), Cognitive Credibility Assessment (CCA) and Assessment Criteria Indicative of Deception (ACID). Meta-analyses are available regarding the first three tools and have shown that all three produce diagnostic verbal cues to deceit (Hartwig et al., 2014; Palena et al., 2021; Vrij, Palena, et al., 2021). Another meta-analysis provided evidence that trained observers perform well above the level of chance in distinguishing between truth tellers and lie tellers if they pay attention to CCA and ACID related cues (Mac Giolla & Luke, 2021).

SUE relates to comparing a statement with evidence (Granhag & Hartwig, 2015). In SUE, interviewers encourage interviewees to discuss their whereabouts during the period of interest without revealing the evidence they possess. Lie tellers’ reluctance to position themselves near the scene of crime (avoid or escape strategy) results, amongst other findings, in more contradictions between statement and evidence amongst lie tellers than amongst truth tellers (Hartwig et al., 2014). If multiple pieces of evidence are available, it is beneficial to introduce them gradually in the interview rather than at once (Dando et al., 2015; Granhag et al., 2013; McDougall & Bull, 2015); see also Sandham et al. (2020).

VA states that truth tellers report more details that an investigator can check than do lie tellers (Nahari, 2018b). Information that can be verified include activities carried out or witnessed by named witnesses or captured on CCTV cameras. Research has found that truth tellers provide more verifiable details than do lie tellers, particularly if the investigator asks the interviewee, when possible, to include in their statements details they can check. This encourages truth tellers more than lie tellers to report verifiable details (Palena et al., 2021).

The CCA interview protocol consists of exhausting the free recall phase of an interview by repeatedly asking interviewees to report all they can remember, albeit in different formats: (a) initial free recall followed by free recalls; (b) after a model statement (a detailed account about a topic unrelated to the event under investigation (Leal et al., 2015; Vrij, Leal, & Fisher, 2018); (c) in reverse order; and (d) while sketching. A key dependent variable is the new information (particularly complications, discussed below) that is added at each stage (Vrij et al., 2015; Vrij, Mann, et al., 2021).

In ACID an initial free recall phase is followed by mnemonics (mental reinstatement of context, recall from other perspective and reverse order recall) to enhance the interviewee’s recall of the event. Truth tellers benefit more from these mnemonics than lie tellers and provide more additional detail (Colwell et al., 2013). In addition, a series of multiple-choice questions are asked in between the different mnemonics. These are questions that lie tellers have not anticipated. They therefore increase cognitive demand on lie tellers, because they must consider what the interviewer knows and does not know about the event (Colwell et al., 2013).

Practitioners often mention that there is a gap between the interview protocols required for using these veracity assessment tools and a typical interview. For these veracity assessment tools to be effective, interviewees should report their experiences in as much detail as possible without interruption (Vrij et al., 2015). The latter is in alignment with how ‘good interview practice’ is advocated by researchers and yields good results in terms of eliciting information, eliciting true confessions and avoiding false confessions (Meissner et al., 2014, 2017; Vrij, Meissner, et al., 2017). Yet, this interview style is often not used. Typically, the initial free recall phase is followed by many questions to which short answers are given (Snook et al., 2012; Vrij et al., 2015). Many transcripts that practitioners show us of interviews conducted in their organisations also follow this format. As a result, we often struggle to give meaningful advice about verbal veracity assessment when reading transcripts of such interviews.

There are several reasons why interviews consisting of many questions and short answers appear to be prevalent. Some interview protocols are designed in this format because there is only a very short time frame to conduct interviews (e.g. 911 interviews and border control interviews). In those cases, interview protocols required for verbal veracity assessment (open-ended recall without interruptions) are difficult to employ. In other settings, there is more time to conduct the interview, and so it would be more suitable for an open-ended recall without interruptions. However, in many of those instances, the interviewee is reluctant to talk. Finally, in some instances, the interviewer is too keen to ask specific questions.

One way to bridge the gap between interview protocols used in verbal veracity assessment (e.g. open-ended questions without interruptions) and a typical interview protocol (e.g. many questions and short answers) is to examine verbal veracity assessment in interview settings in which respondents do not provide detailed narrative statements. Researchers could start collaborating with practitioners and either they could examine whether existing veracity assessment tools could be made suitable for the interview protocols that practitioners use or, alternatively, researchers could design entirely new veracity assessment tools to meet the briefer answers that respondents current give. Research into which verbal cues are diagnostic to deception in interviews consisting of brief answers is scarce, but see Ormerod and Dando (2015) for an exception.

### The most diagnostic verbal cue to deceit (total details) is unsuitable for lie detection purposes

The total amount of information people provide (total details) appears to be the most diagnostic cue to distinguish truth tellers from lie tellers, with truth tellers typically providing more details than lie tellers (Amado et al., 2016). Total details also seems to be the most frequently investigated verbal cue in deception research (Amado et al., 2016; Vrij, 2008) and has also been suggested as a potentially effective cue for lie detection (Verschuere, Bogaard, et al., 2021; Verschuere, Lin, et al., 2021).

We think that total details is unsuitable for lie detection purposes. First, it is impractical. In research, details are typically operationalised by transcribing the interviews and counting the number of units of unique information. Transcribing interviews and coding these transcripts refers to post-interview deception detection, a method that also can be used in some real-life situations. However, in other real-life investigations, practitioners cannot use this operationalisation, because there is urgency to make a veracity decision (e.g. border control interviews) or because practitioners do not have the resources available to transcribe interviews and read transcripts (e.g. many police–suspect interviews). In such situations real-time deception detection is the preferred method. Investigators have two options if they want to examine total details when transcribing the interviews is not available. First, they could count the number of details an interviewee provides during interviews in real time. As we explained above this is an impossible task. Second, they could estimate the number of details someone provides. Experiments in which coding objective details (frequency of occurrence) and coding subjective details (7-point Likert scale) are compared are scarce, and we are aware of only two such experiments. Shaw et al. (2014) found a high correlation (*r* = .67) between the two types of coding. Moreover, the typical finding for details – truth tellers reported more details than lie tellers – emerged in both types of coding. In unpublished work, Verschuere, Lin, et al. (2021) found that observers instructed to make decisions based on detailedness when evaluating written statements were very accurate in distinguishing truth tellers from lie tellers. This accuracy outperformed the accuracy based on objective coding of this cue. This suggests that a subjective estimation of details could be an adequate alternative to the objective measurement. However, in both experiments the estimations were carried out by coders (Shaw et al., 2014) and observers (Verschuere, Lin, et al., 2021) based on the transcripts of the interview and not by the interviewers themselves during the interviews. It is therefore premature to suggest that interviewers can estimate the actual number of details reported in interviews in real time. Future research should examine this.

Second, total details is in all likelihood vulnerable to countermeasures: lie tellers’ efforts, after learning how a lie detection tool works, to adjust their responses so that they come across as truth tellers when the tool is used. All lie tellers need to do to appear honest in interviews where investigators use total details as a cue to deceit is to provide details. Lie tellers are surely capable of doing just that. To detect deceit in such interviews, investigators have no choice other than to examine the types of detail that interviewees report.

Rather than examining verbal cues that practitioners cannot count in real time and are vulnerable to countermeasures (e.g. total details), researchers could provide practitioners with verbal cues that can be counted in real time and are more resistant to countermeasures. Three examples are: complications, verifiable sources and plausibility. Complications, which are uttered more by truth tellers than lie tellers (Vrij, Palena, et al., 2021), are added clusters of details that make the story more complicated (e.g. ‘Initially we did not see our friend, as he was waiting at a different entrance’). Because complications are clusters of details, there are fewer complications than there are details. The example above contains seven details (underlined) but one complication. From workshops we give about lie detection we know that practitioners can count complications in real time, and research has shown that they frequently occur in truthful stories even when these truthful stories describe a short period of time (Vrij, Palena, et al., 2021). Complications are also to some extent resistant to countermeasures. In an experiment in which lie tellers were informed about the relationship between complications and deception, they still reported fewer countermeasures than did truth tellers (Vrij, Leal, Fisher, et al., 2020).

Verifiable sources are derived from the Verifiability Approach. Take as example the following sentence: ‘In the afternoon, I went shopping with my friend Fred and bought trousers and two pairs of shoes’. The Verifiability Approach recommends counting the number of verifiable details in a statement (nine in the example) but, like total details, they cannot be counted in real time. However, someone can count the number of verifiable sources in a statement (one in the example above ‘Fred’). Truth tellers typically report more verifiable sources than do lie tellers (Leal et al., 2018; Vrij et al., 2019; Vrij, Mann, et al., 2021). This variable also seems to be resistant to countermeasures. In fact, informing truth tellers and lie tellers about the working of the Verifiability Approach (i.e. informing interviewees that the investigator would like to hear details she or he can check) increased the difference between truth tellers and lie tellers in reporting verifiable details, because it made truth tellers report more additional verifiable details than lie tellers (Nahari et al., 2014).

Plausibility, a cue to truthfulness, is perhaps the verbal cue that can be measured most easily in real time. It can be defined as *how likely is it that the activities happened in the way described* (Leal et al., 2019, p. 278). Research has shown that plausibility distinguishes truth tellers from lie tellers better than most other verbal indicators (Vrij, Deeb, et al., 2021). It is widely used as a veracity assessment tool, for example, in asylum seekers interviews in Australia and the European Union (Luker, 2013; UNHCR, 2013). Yet researchers appear sceptical in examining plausibility and in recommending its use to practitioners (Vrij, Deeb, et al., 2021). Their problem is the subjective nature of the cue. Unlike most other verbal cues, it cannot be operationalised as frequency of occurrences in a statement. Instead, it is a global impression of that statement. However, research has shown that it is possible to measure plausibility reliably (Vrij, Deeb, et al., 2021). In addition, re-analysing five data sets has shown that plausibility can be predicted by two cues that can be measured objectively by counting their frequency of occurrence: Complications and verifiable sources, explaining almost 40% of the variance (Vrij, Deeb, et al., 2021). In addition to these cues, practitioners can take the context into account and judge a statement in terms of what is conventional or reasonable in a given situation (unconventional or unreasonable activities are considered implausible). This context-based lie detection has shown good potential (Blair et al., 2010). Given that plausibility would be a convenient cue for practitioners to use and that research has given concrete instructions about what to do, we recommend researchers no longer ignore it. Perhaps they can explore the benefits and costs of plausibility judgements and address the subjectivity issue. We are not aware of countermeasures research regarding plausibility. We do not think that lie tellers will be able to use successful countermeasures if they try to do so. Arguably, it is easier for lie tellers to successfully employ countermeasures if they are given concrete instructions what to do to come across as honest. Such concrete information is not available regarding plausibility.

### Predominantly cues to truthfulness do exist

A distinction can be made between cues to deceit and cues to truthfulness. Cues to deceit are cues that lie tellers display more frequently than truth tellers, and cues to truthfulness are cues that truth tellers display more frequently than lie tellers. Most nonverbal cues are cues to deceit. For example, it is assumed that lie tellers compared to truth tellers show more gaze aversion, more fidgeting and more pauses (Strömwall et al., 2004). Practitioners who attempt to detect deceit through observing nonverbal behaviour are thus used to looking for cues to deceit.

In contrast, most verbal indicators of deception are cues to truthfulness. Total number of details, for example, is a cue to truthfulness. Criteria-Based Content Analysis, a widely researched list of verbal veracity indicators, consists of 19 verbal criteria, and they are all cues to truthfulness (Amado et al., 2016). Reality Monitoring, another frequently used list of verbal veracity indicators (Masip et al., 2005; Sporer, 2004), consists of eight criteria, and all but one are cues to truthfulness. The exception is the cue ‘Cognitive operations’, which lie tellers are predicted to use more frequently than truth tellers (Masip et al., 2005; Vrij, 2008). Researchers disagree about how to operationalise cognitive operations (Vrij, 2008), and it does not discriminate between truth tellers and lie tellers (Masip et al., 2005; Vrij, 2008).

The Verifiability Approach states that truth tellers report more details that an investigator can check than do lie tellers (Nahari, 2018b), and research has supported this assumption (Palena et al., 2021). This makes verifiable details a cue to truthfulness. However, the opposite, that lie tellers report more unverifiable details than truth tellers, is not true (Palena et al., 2021). Furthermore, the Verifiability Approach does not predict that such a difference will occur. From the verbal veracity tools available to date, only SUE focuses on cues to deceit, statement–evidence inconsistency and within-statement inconsistency (Hartwig et al., 2014).

Verbal lie detection may become more popular amongst practitioners if it does what practitioners typically do: pay attention to cues to deceit. In most daily-life situations, people are generally truthful (Levine, 2014). This makes (a) assuming that a statement is true (truth default) and (b) being inclined to evaluate a statement as true (true bias) successful lie detection strategies (Levine, 2014). However, in interviews the chance that suspects lie is real, and suspicion is often actively triggered. Attempting to detect deceit then becomes a priority, and paying attention to cues to deceit sounds reasonable. It makes sense that when detecting lies practitioners would like to obtain direct evidence (e.g. cues to deceit present) rather than indirect evidence (e.g. cues to truthfulness absent). Direct evidence does not require any reasoning to draw a conclusion based on the evidence whereas indirect evidence requires that an inference be made between the evidence and conclusion to be drawn from it.

In fact, a verbal veracity tool (or any veracity tool) that measures a mixture of cues to truthfulness and cues to deceit (combining two sources of direct evidence) is most desirable. Someone would be able to say with much more confidence that someone is lying when not only cues to deceit are present but also when cues to truthfulness are absent, and, vice versa, someone would be able to say with much more confidence that someone is telling the truth when not only cues to truthfulness are present but also when cues to deceit are absent.

Researchers have started recently to examine two verbal cues to deceit that lie tellers appear to report more frequently than truth tellers (Vrij, Palena, et al., 2021): common knowledge details and self-handicapping strategies. Common knowledge details refer to strongly invoked stereotypical information about events (‘The event had an Oscars theme, so everybody was dressed up’). Self-handicapping strategies refer to justifications as to why someone chooses not to provide information (‘There isn’t much to say about the actual bungee jump as it took only a few moments’). Like complications and verifiable sources, these two cues can be counted in real time (Vrij, Palena, et al., 2021).

Common knowledge details and self-handicapping strategies are frequently examined together with complications (a cue to truthfulness) but they are not as strongly related to veracity as complications (Vrij, Palena, et al., 2021). Truth tellers also produce statements that include common knowledge details, particularly when they do not see the importance to report an event in much detail. Self-handicapping strategies do not occur frequently, which makes them of limited use to identify deception. In addition, unlike the occurrences of complications amongst truth tellers, the occurrences of common knowledge details and self-handicapping strategies amongst lie tellers may depend on the type of scenario they discuss. They are typically examined in a ‘travel’ scenario where interviewees report a trip they allegedly have made in the last twelve months (Vrij, Palena, et al., 2021). Travelling is arguably a somewhat scripted activity, which makes common knowledge details more likely to occur (‘We visited the famous cathedral. After that we strolled over the market and in the evening we had dinner in a Chinese restaurant’). When the trip occurred not recently, lie tellers have a good opportunity to include self-handicapping strategies (‘I cannot remember which restaurants we visited in the evenings; we went there three months ago’). The situation is different when someone describes a unique event that has just happened. Complications are perhaps not affected much by recency (Vrij, Palena, et al., 2021) but common knowledge details are perhaps more likely to occur when someone describes a somewhat scripted event rather than a unique event; self-handicapping strategies are more likely to occur when someone describes an event after a delay rather than immediately (Vrij, Palena, et al., 2021). In summary, common knowledge details and self-handicapping strategies have shortcomings as verbal cues to deception. It is important for practitioners that researchers search for verbal cues to deceit that are more diagnostic than common knowledge details and self-handicapping strategies (see also Nahari et al., 2019).

### Cut-off points do not exist

The verbal information that an interviewee provides depends on his/her personality and the situation. Individuals differ in terms of how much information they spontaneously recall (Nahari & Pazuelo, 2015; Nahari & Vrij, 2014). For example, participants high on fantasy proneness produced statements that were richer in detail than participants low on fantasy proneness (Merckelbach, 2004), although Boskovic et al. (2021) could not replicate this finding. Richness in detail was positively correlated with social adroitness and self-monitoring, but negatively correlated with social anxiety (Vrij et al., 2002, 2004).

The amount of verbal information someone provides also depends on the situation. Some factors are under control of the interviewer (system variables; Wells, 1978): appropriate questions resulting in more information than inappropriate questions (Oxburgh et al., 2012); and interview protocols such as the Cognitive Interview resulting in more information than standard type of interviews (Memon et al., 2010). Other factors are beyond the interviewer’s control (estimator variables; Wells, 1978), such as the extent to which an experience was rich in detail (Vrij, 2008), how much attention the interviewee paid to the to-be-discussed event (Harvey et al., 2017) and the time delay between the event and interview (Harvey et al., 2017; Nahari, 2018a). As these factors vary markedly from one situation to another, no firm cut-offs can be used in any one situation.

In experimental research truth tellers and lie tellers are compared at a group level. In these experiments, the only difference between truth tellers and lie tellers is the difference in veracity status, because individual and situational differences are controlled for. This does not reflect real life where practitioners must make veracity assessments based on an individual case in which individual and situational differences play an important part. Based on the number of (verifiable) details that someone reports, practitioners cannot conclude that someone is telling the truth or lying because the score will be influenced not only by the veracity status of the interviewee but also by his/her personality, the situation and a host of other factors; see Nahari et al. (2019).

This absence of cut-off points does not only refer to verbal lie detection; it also applies to nonverbal lie detection. It does not seem to bother practitioners as much in nonverbal lie detection as in verbal lie detection. A possible explanation is that because practitioners focus on lie detection rather than truth detection, they easily accept the nonverbal signs of deceit as support for their view that someone is lying.

To control for individual and situational differences, *verbal baselining* could be used. Effective baselining controls for individual differences (compare different parts of a statement within the same person) and for situational differences (compare different parts of the statement in which the person discusses the same event). Most verbal baselining research to date controls only for individual differences, which yields limited success (Bogaard et al., 2022; Palena et al., 2018; Verigin et al., 2021).

In effective verbal baselining the same person talks about the same event in different formats. For example, the interviewee could first be asked to recall all she or he remembers about the event. After this initial recall, she or he could be asked to listen to a model statement and then be asked again to report the experienced event. Research has shown that the model statement results in truth tellers adding more complications to their stories than lie tellers (Deeb et al., 2020; Vrij, Leal, et al., 2017; Vrij, Mann, et al., 2020). An alternative technique is, after the initial recall, to ask interviewees to report the event again but this time to sketch their experiences while narrating. This also leads to more complications amongst truth tellers than amongst lie tellers (Vrij, Leal, et al., 2018; Vrij, Mann, et al., 2021; Vrij, Mann, et al., 2020). Another alternative technique is, after an initial recall, to ask interviewees to report the event again, but this time to include sources the investigator can check. This leads to truth tellers reporting more verifiable sources than lie tellers (Vrij, Mann, et al., 2021).

Letting the same person talk about the same event in different formats controls for individual and situational differences and is therefore a step forward in solving the cut-off point problem. It does not resolve the cut-off point problem entirely, because it is still unknown how many complications or how many verifiable sources an interviewee should report to be classified as a truth teller. For cut-off points to work, truth tellers and lie tellers should report different verbal cues rather than truth tellers and lie tellers reporting the same cues but in different frequency (lie tellers report fewer complications and fewer verifiable sources than truth tellers).

Solving the cut-off score issue is challenging in lie detection, and a solution will not be easy to find. A possible solution is to examine the effect of having an ‘inconclusive outcome category’ on accuracy rates, as used in polygraph examinations (Kleiner, 2002). That is, in verbal lie detection interviewees are classified as either truth tellers or lie tellers. In contrast, in polygraph examination a third category is introduced for cases with an uncertain polygraph outcome: inconclusives. It is worth examining whether the introduction of inconclusive outcomes in verbal deception research will improve the accuracy rates of classifying truth tellers and lie tellers.

### Different types of lie may result in different verbal indicators

Psychologists have made a distinction between four types of lie (DePaulo et al., 1996; Leins et al., 2013). Outright lies are lies in which the information that is conveyed is totally false. Exaggerations are distortions of the truth, such as overstating or understating facts. Embedded lies are lies in which the false information is incorporated in an otherwise truthful story; and omissions are lies by deliberately omitting relevant information. Telling different types of lie may result in different cognitive processes (Sporer & Schwandt, 2006). For example, telling an outright lie is more mentally taxing than omitting information (Sporer & Schwandt, 2006). Subsequently, some verbal cues may be specifically associated with a certain type of lie. However, research examining this is largely absent because most research focuses on total fabrications (Vrij, 2008), but see Verigin et al. (2020) for an outright lies – embedded lies comparison.

From a practical point of view, establishing which verbal cues are specifically associated with which type of lie is of little use because practitioners will not know what type of lie an interviewee may tell. It is thus more relevant to focus on similarities between the different types of lie rather than on their differences. Leal and colleagues did exactly that (Leal et al., 2020). They argued that when lying through omitting information, where the information lie tellers provide is entirely truthful, lie tellers may use the same strategy as they use when telling total falsehoods: ‘to keep it simple’. Leal et al. (2020) thus hypothesised that even in lying through omissions scenarios, lie tellers still may include fewer details and fewer complications in their accounts than truth tellers. No difference emerged for total details but lie tellers indeed included fewer complications in their statements than truth tellers. This finding was replicated in a second lying through omitting information experiment (Leal et al., 2021). These findings suggest that complications could be a diagnostic veracity indicator across different types of lie.

### People from different cultures may show different verbal cues to deceit

Not only could verbal veracity indicators differ per type of lie, they may also be culturally determined. Cross-cultural research examining verbal veracity cues is sparse, but the limited work reveals that culturally defined verbal veracity indicators do exist (Taylor et al., 2017; Taylor et al., 2014). Taylor and colleagues (2014) examined verbal veracity cues amongst several cultural groups: Arab, Pakistani, North African, South Asian, White British and White European. Several culturally specific veracity cues emerged. For example, the use of negations (e.g. denials) was a cue to deceit in Arab and Pakistani populations, but not a veracity indicator in White British and North African populations, and the use of spatial information was a cue to deceit in North African and Pakistani populations but a cue to truthfulness in Arab and White British populations.

The modern world, with widespread travel, means that practitioners frequently interview individuals belonging to countries and cultures other than their own. It will make the already difficult task of lie detection even more difficult if investigators have to take a culture into account when assessing veracity based on speech content. The most straightforward solution is to focus on verbal cues that are culturally independent. In the, yet scarce, research in this area some of such cues have emerged. Although people from individualistic cultures (Western cultures) tend to provide more information than people from collectivistic cultures (non-Western cultures; Anakwah et al., 2020; Hope et al., 2022; Wang et al., 2017), the finding that truth tellers report more details than lie tellers has been replicated in various non-Western cultures, including in Arab, Chinese, Russian and South Korean populations (Leal et al., 2018; Vrij, Leal, Mann, et al., 2020). The findings that truth tellers report more complications and fewer common knowledge details and self-handicapping strategies than lie tellers has been found outside the Western world in Hispanic, Russian and South Korean populations (Vrij & Vrij, 2020). We encourage researchers to continue searching for culturally independent verbal veracity indicators.

## Conclusion

Although many researchers made the shift from nonverbal to verbal lie detection, most practitioners have yet to follow suit. In this article we addressed concerns practitioners raised about verbal lie detection. We believe that simply telling practitioners that verbal lie detection is more accurate than nonverbal lie detection and that many of their concerns also apply to nonverbal lie detection will not convince practitioners to make the change. Many concerns practitioners raised are caused by gaps in research. To make verbal lie detection more acceptable amongst practitioners researchers should address these limitations by examining: (a) verbal veracity assessment tools in interviews that consist of many questions and short answers; (b) verbal cues that can be estimated in real time and are not vulnerable to countermeasures; (c) verbal cues to deceit in addition to cues to truthfulness; and verbal veracity cues that occur independently from (d) type of lie and (e) cultural background of the interviewee.

There is a lot to do for researchers. The discrepancies between how practitioners work and the way verbal lie detection functions makes it not straightforward to encourage practitioners to make the switch from nonverbal to verbal lie detection. Instead, it requires creative solutions. We hope that this article provides researchers with ideas for such solutions.

## Ethical standards

### Declaration of conflicts of interest

Aldert Vrij has declared no conflicts of interest.

Ronald P. Fisher has declared no conflicts of interest.

Sharon Leal has declared no conflicts of interest.

### Ethical approval

This article does not contain any studies with human participants or animals performed by any of the authors.
